# Effect of astaxanthin in type-2 diabetes -induced APPxhQC transgenic and NTG mice

**DOI:** 10.1016/j.molmet.2024.101959

**Published:** 2024-05-17

**Authors:** Joshua Adekunle Babalola, Anika Stracke, Tina Loeffler, Irene Schilcher, Sideromenos Spyridon, Stefanie Flunkert, Joerg Neddens, Ake Lignell, Manuela Prokesch, Ute Pazenboeck, Herbert Strobl, Jelena Tadic, Gerd Leitinger, Achim Lass, Birgit Hutter-Paier, Gerald Hoefler

**Affiliations:** 1 Diagnostic and Research Institute of Pathology Medical University of Graz, Graz, Austria; 2 Division of Immunology and Pathophysiology, Otto Loewi Research Center, Medical University of Graz, Austria; 3 QPS Austria GmbH, Grambach, Austria; 4 Medical University of Vienna, Vienna, Austria; 5 AstaReal AB, Sweden; 6 Institute of Molecular Biosciences, University of Graz, Austria; 7 Division of Cell Biology, Histology and Embryology, Gottfried Schatz Research Center, Medical University of Graz, Austria

**Keywords:** Alzheimer's disease, Type 2 diabetes, Pyroglutamylation, Metabolic perturbation, Astaxanthin

## Abstract

**Objectives:**

Aggregation and misfolding of amyloid beta (Aβ) and tau proteins, suggested to arise from post-translational modification processes, are thought to be the main cause of Alzheimer's disease (AD). Additionally, a plethora of evidence exists that links metabolic dysfunctions such as obesity, type 2 diabetes (T2D), and dyslipidemia to the pathogenesis of AD. We thus investigated the combinatory effect of T2D and human glutaminyl cyclase activity (pyroglutamylation), on the pathology of AD and whether astaxanthin (ASX) treatment ameliorates accompanying pathophysiological manifestations.

**Methods:**

Male transgenic AD mice, APPxhQC, expressing human APP751 with the Swedish and the London mutation and human glutaminyl cyclase (hQC) enzyme and their non-transgenic (NTG) littermates were used. Both APPxhQC and NTG mice were allocated to 3 groups, control, T2D-control, and T2D-ASX. Mice were fed control or high fat diet ± ASX for 13 weeks starting at an age of 11–12 months. High fat diet fed mice were further treated with streptozocin for T2D induction. Effects of genotype, T2D induction, and ASX treatment were evaluated by analysing glycemic readouts, lipid concentration, Aβ deposition, hippocampus-dependent cognitive function and nutrient sensing using immunosorbent assay, ELISA-based assays, western blotting, immunofluorescence staining, and behavioral testing via Morris water maze (MWM), respectively.

**Results:**

APPxhQC mice presented a higher glucose sensitivity compared to NTG mice. T2D-induced brain dysfunction was more severe in NTG compared to the APPxhQC mice. T2D induction impaired memory functions while increasing hepatic LC3B, ABCA1, and p65 levels in NTG mice. T2D induction resulted in a progressive shift of Aβ from the soluble to insoluble form in APPxhQC mice. ASX treatment reversed T2D-induced memory dysfunction in NTG mice and in parallel increased hepatic pAKT while decreasing p65 and increasing cerebral p-S6rp and p65 levels. ASX treatment reduced soluble Aβ38 and Aβ40 and insoluble Aβ40 levels in T2D-induced APPxhQC mice.

**Conclusions:**

We demonstrate that T2D induction in APPxhQC mice poses additional risk for AD pathology as seen by increased Aβ deposition. Although ASX treatment reduced Aβ expression in T2D-induced APPxhQC mice and rescued T2D-induced memory impairment in NTG mice, ASX treatment alone may not be effective in cases of T2D comorbidity and AD.

## Highlights

• APPxhQC mice showed better glucose tolerance.
• T2D-induced APPxhQC mice present a shift from soluble to insoluble Aβ isoforms in the brain.
• ASX treatment rescues hippocampus-dependent cognitive impairment in T2D-induced NTG mice.
• ASX treatment reduces soluble and insoluble Aβ isoforms in T2D-induced APPxhQC mice.

## 1 Introduction

Alzheimer's disease (AD) is a neurodegenerative disorder with multiple aetiologies that is characterized by pathophysiological manifestations such as dysfunction of the neurovascular unit [[1], [2], [3], [4]], loss of memory function [5], tau-related lesions in neurons termed neurofibrillary tangles [6,7], synaptic degeneration [8,9], neuroinflammation [[10], [11], [12], [13]], metabolic perturbation [14,15], neuronal cell death [16] and, most importantly, aggregation of amyloid beta (Aβ) [17].

The proteolytic processing of the Aβ protein precursor (APP) by β- and γ-secretases results in the generation of Aβ peptides [18]. Aβ-associated plaques in the brain of AD patients have been reported to contain a diverse mixture of Aβ peptides [19]. In addition to the main Aβ species Aβ_40_ and Aβ_42_, other variants originate from post-translational modifications such as truncation, racemization, isomerization, phosphorylation, metal-induced oxidation, and pyroglutamination [[20], [21], [22], [23], [24], [25], [26], [27], [28], [29], [30], [31], [32], [33]].

N-terminal truncation and subsequent cyclization of Aβ peptides leads to the formation of pyroglutamate (pGlu)-Aβ peptides [20,34,35], which constitute the majority of Aβ deposits in sporadic and familial AD [20,22,36,37]. pGlu-Aβ peptides are present in senile plaques and vascular amyloid deposits [20,21,[38], [39], [40]]. Pyroglutamination of Aβ peptides is catalysed by glutaminyl cyclase (QC) *in vitro* [41] as well as *in vivo* [[42], [43], [44], [45]]. In transgenic mouse models of AD, the presence of pGlu-Aβ peptides leads to protein aggregation and deposition, neurodegeneration, gliosis, and impairment of learning and memory [46]. Genetic ablation or pharmacological inhibition of QC in mouse and fruit fly models result in reduced pGlu Aβ peptide production and improved performance in cognitive tasks [44,47,48].

Although age is the greatest pre-disposing risk factor for AD [49], sex and modifiable lifestyle factors like type 2 diabetes (T2D) have also been reported to increase the risk of developing AD [50].

T2D is a global epidemic with the diabetic population projected to reach 1.2 billion by the year 2050 [51]. Substantial evidence is available that links metabolic dysfunctions such as obesity, T2D, and dyslipidemia to the pathology of AD. The comorbid association between T2D and AD is strong - the risk of developing AD has been found to be 65% higher among diabetic patients than in non-diabetic controls [52]. In a large community study, the rate of diabetes was found to be 35% among AD patients. About 46% of AD patients were glucose intolerant, a well-known precursor for T2D [52]. Insulin resistance is associated with many chronic diseases such as T2D, obesity and AD [53]. Peripheral insulin resistance and in CNS is present in about 35% of people diagnosed with AD [54]. Insulin resistance in the CNS is entirely different from peripheral insulin resistance [53]. The receptors of the CNS have been found not to participate in the classic negative feedback loop between glucose and insulin levels. Hence, deficient insulin activity in the CNS likely has no effect on blood insulin levels. Cerebral insulin resistance has been reported in individuals with peripheral insulin resistance, in aged individuals, as well as in AD patients [55]. In 2012, Talbot and others showed that insulin resistance in the periphery does not automatically confer CNS insulin resistance. They reported that CNS insulin receptor resistance was present in most patients diagnosed with AD even in the absence of a peripheral diabetic state. In addition, in a *post-mortem* study, they showed an impaired response to insulin in different brain regions including the cerebellar cortex and hippocampal formation of AD patients compared to controls who did not have any evidence of peripheral insulin resistance. This landmark study suggests that insulin resistance in the CNS is independent of peripheral insulin resistance [55].

The benefit of the currently approved anti-amyloid monoclonal antibodies, Aducanumab and Lecanemab, is still discussed controversially, and they are reported to cause considerable side effects [[56], [57], [58], [59]]. Acetylcholinesterase inhibitors (AChEI) (tacrine, galantamine, rivastigmine and donepezil) [[60], [61], [62]] and memantine, a low affinity NMDA receptor antagonist [63,64] are reported to cause only symptomatic relief. Furthermore, anti-tau antibodies have failed in clinical trials [[65], [66], [67], [68], [69], [70]]. lt is therefore important to develop new drugs and/or repurpose approved drugs that target multiple disease pathologies, are relatively inexpensive and are easily accessible.

Astaxanthin (ASX) is a lipid-soluble keto-carotenoid synthesized by several microorganisms and various types of marine organisms [71]. It is one of the most potent antioxidants found in nature (Ambati et al., 2014). ASX has been reported to exhibit pharmacological activities, such as immunomodulatory, antioxidative, anti-apoptotic, anti-inflammatory, anti-cancerous, and anti-diabetic effects [[72], [73], [74], [75]]. The neuroprotective effects of ASX have been corroborated previously *in vitro* [76,77], *in vivo* [[78], [79], [80]] and in clinical studies [81]. Due to ASX's strong antioxidative and anti-inflammatory properties, it has potential to ameliorate a wide range of brain disorders such as AD, Parkinson's disease, depression, brain stroke, and autism spectrum disorders [82]. However, the therapeutic potential of ASX for the co-morbidity of T2D and AD is unclear. In this study we aimed to investigate the combinatory effects of human glutaminyl cyclase activity (pyroglutamylation) and metabolic dysfunction in AD pathology by inducing T2D in the pGlu-Aβ-expressing APPxhQC mouse model. Furthermore, we evaluated the therapeutic potential of ASX for the treatment of pathophysiological manifestations associated with a combination of metabolic impairment and pyroglutamylation. Analyses focussed on glycemic readouts, Aβ metabolism, spatial cognitive impairment and nutrient-sensing signalling.

## 2 Materials and methods

### 2.1 Animals & housing

All mice used in this study were bred in the animal facility of QPS Austria GmbH. Animal breeding and experiments were approved by the Styrian government, Austria (approval number: ABT13-78Jo365-2022). Animals were housed in individually ventilated cages on standardized rodent beddings (Rettenmaier & Söhne, Vienna, Austria). The room temperature was maintained between 20 and 24 °C; the relative humidity was maintained between 45 and 65%. Animals were housed under a constant light-cycle (12-hour light/dark). Male APPxhQC, crossbreds of APP_SL_ and hQC mice, and non-transgenic (NTG) littermates were used in this study. APP_SL_ mice express human APP751 with the Swedish and the London mutation on a C57Bl/6RccHsd background. hQC mice express human glutaminyl cyclase (hQC) enzyme on a B6CBAF1/J background. The crossbreeding results in an increased generation of N-terminally modified pGlu Aβ peptides [44,83]. Age-matched male littermates were used as controls.

### 2.2 Feeding, experimental model and timeline

Twenty-two APPxhQC mice and NTG littermates, aged 11–12 months, were both allocated to 3 groups with 3–4 animals per group: Control group, T2D-Control group, and T2D-ASX group. Mice in the control groups were fed control diet (CD), containing 10 kJ% fat, 7% sucrose (Supplementary Table 1, ssniff® diets, Germany). Animals of the T2D-Control group were fed high fat diet (HFD), containing 95% (60 kJ% fat) + 5% maltodextrin placebo diet (Supplementary Table 2, ssniff® diets, Germany) with daily injection of 40 mg/kg streptozocin for 3 days, while animals of the T2D-ASX groups were given HFD containing 95% (60 kJ% fat) + 5% NOVASTA containing 5% pure ASX (AstaReal, Sweden), a feed ingredient containing natural astaxanthin whole-algae, alongside daily injection of 40 mg/kg streptozocin (Sigma–Aldrich, Germany) for 3 days. The T2D induction model is an Insulinopenic model due to the toxic effect of Streptozocin but also expected to present insulin resistance attributable to HFD. Mice in the T2D-ASX groups consumed approximately 137 mg/kg/per day of pure ASX. Dietary treatment lasted for 13 weeks. Body weights were recorded, and food was changed weekly. After 3 weeks of HFD feeding, T2D-Control groups and T2D-ASX groups were intraperitoneally treated with 40 mg/kg streptozocin once daily for 3 days, while control groups were injected with 50 nM sodium citrate buffer. The intraperitoneal glucose tolerance test (ipGTT) was conducted prior to the feeding start and in treatment week 12. The Morris water maze (MWM) was performed in treatment week 13. Plasma and tissues were harvested at the end of treatment week 13.

### 2.3 Intraperitoneal glucose tolerance test (ipGTT)

Mice were fasted 5 h before tail veins of all animals were punctured and blood taken. Basal blood glucose concentrations were measured using the glucose analyzer (Nova StatStrip Xpress®-i, Nova Biomedical, Waltham, MA, USA). Subsequently, a single dose of a 20% glucose solution was intraperitoneally injected (2 g/kg body weight) before additional blood glucose concentration measurements were performed 15-, 30-, 60- and 120-minutes post injection [84].

### 2.4 Intraperitoneal insulin tolerance test (ipGTT)

Mice were fasted for 5 h before fasting blood glucose levels (“0”) were measured from tail vein blood. A single dose of dose of 0.75 U human insulin peptide per kilogram (kg) of body weight was injected intraperitoneally before additional blood glucose concentration measurements were taken after 15-, 30-min post injection using the glucose analyzer (Nova StatStrip Xpress®-i) [84]. Mice were closely observed for 30 min and in case blood glucose dropped below 20 mg/dl, or animal appeared hypoglycemic (tremor, heightened anxiety or apathy, paresthesia, loss of consciousness and, rarely, convulsions), 300 μl of 20% glucose solution was administered.

### 2.5 Morris water maze

The Morris water maze test was conducted in a circular 120 cm diameter pool filled with opaque water. The maze was placed in a sound-proof room with cues on each wall. The training paradigm consisted of six consecutive days with two trials per day. During the training sessions, mice were allowed to swim for 60 s (s) per trial to locate the hidden platform that was submerged 1 cm below the water's surface. The time spent to find the hidden platform was recorded and the average of the two trials per day was determined. Once the mouse found the platform it was left on the platform for 15 s. If the mouse did not find the platform it was guided to the platform and left there for 10 s. One day after the last training, the platform was removed, and a probe trial was performed to assess short-term spatial memory. Mice were tested to find the platform from the opposite quadrant for 60 s. For the quantification of the escape latency (the time [s] to find the hidden platform), the pathway (the length of the trajectory [meters] to reach the target), the number of target zone crossings and the abidance in the target quadrant [s] in the PT, a computerized video tracking system (Noldus Ethovision XT 14, Noldus, Wageningen, the Netherlands) was used.

### 2.6 Tissue sampling

After 13 weeks of feeding with HFD or control diet, mice were fasted for 5 h, tested in the ipGTT, and sacrificed by pentobarbital injection (600 mg/kg). The thorax was opened, and blood was collected by heart puncture, 50 μl of whole blood was transferred into K2-EDTA tubes (Greiner Bio-One International, Vienna, Austria) and the rest divided and transferred into two vials using K2-EDTA for plasma and serum gel clotting activator microtube (SARSTEDT, Nümbrecht, Germany) for serum. After incubation for at least 20–60 min at RT, serum was prepared from blood samples by centrifugation for 5 min at 10,000×*g* and supernatant was collected. Plasma was sampled into K2-EDTA (Potassium ethylenediaminetetraacetic acid) tubes. The tube was inverted thoroughly to facilitate homogeneous distribution of the EDTA and to prevent clotting. Blood samples were centrifuged at 3,000×*g* for 10 min at RT. Serum and plasma was transferred to pre-labelled Eppendorf LoBind tubes and stored at −80 °C until biochemical analysis. Additionally, brain and liver were sampled for further investigation. The right brain hemispheres and left liver lobes were fixed by immersion in freshly prepared 4% PFA for 2 h at room temperature. Thereafter, right brain hemispheres were transferred to 15% sucrose in PBS solution until sunk (at 4 °C) to ensure cryoprotection. Afterwards, fixed brain hemispheres and liver lobes were frozen using OCT medium in cryo molds in dry-ice cooled isopentane and stored at −80 °C until used for histological analysis. The left-brain hemispheres and rest of the liver used for biochemical evaluation were weighed before being immediately frozen on dry ice and stored at −80 °C.

### 2.7 Measurement of plasma lipid and serum liver enzymes

Determination of cholesterol, triglyceride, aspartate aminotransferase (AST) and alanine aminotransferase (ALT) concentrations were performed using the CHOL2 (item # 03039773190), TRIGL (item # 20767107322), ASTLP (item # 04467493190) and ALTLP (item # 04467388190) reagents (Roche, Germany). Absorbance was measured at 700/505 nm for plasma cholesterol as well as triglycerides and 700/340 nm for serum AST as well as ALT using Roche Cobas 6000 Chemistry Analyzer (Roche Diagnostics, Indianapolis, IN 46250).

### 2.8 Measurement of blood Hb1Ac and plasma insulin

Determination of the average blood glucose level over the past 8–12 weeks of the treatment phase was performed using the mouse HbA1c ELISA kit 80310 (Crystal Chem, Zaandam, Netherlands). The Ultra-Sensitive Mouse Insulin ELISA Kit 90080 (Crystal Chem, Zaandam, Netherlands) was used for analysis of insulin levels in plasma samples.

### 2.9 Extraction of DEA-soluble and FA-insoluble Aβ fractions, Aβ_38_, Aβ_40_, and Aβ_42_ evaluation and pGlu3-Aβ_42_ measurement

Left brain samples were homogenized in tissue homogenization buffer (THB; 1 M Tris, 100 mM EDTA, 40 mM EGTA containing protease and phosphatase inhibitor). For extraction of non-plaque-associated proteins, THB homogenates were mixed with 1-part diethylamine (DEA) solution (0.4% DEA, 100 mM NaCl). The mixture was centrifuged for 106 min at 20,817 g at 4 °C. The supernatants were neutralized with 1/10 of the volume 0.5 M Tris–HCl, pH 6.8, briefly vortexed and stored at −20 °C. For extraction of plaque-associated proteins, THB homogenates were mixed with 2.2 parts cold formic acid (FA), sonicated for 30 s on ice and centrifuged for 106 min at 20,817×*g* at 4 °C. The supernatant was diluted with 19 parts FA Neutralization Solution (1 M Tris, 0.5 M Na_2_HPO_4_, 0.05% NaN_3_), briefly vortexed and stored at −20 °C. Aβ_38_, Aβ_40_ and Aβ_42_ were measured in the DEA and FA fractions from left-brain homogenates using an immunosorbent assay (Aβ Peptide Panel 1 (6E10); K15200E-2; Meso Scale Discovery, Meso Scale Diagnostics, Maryland, USA) according to the instructions of the manufacturer. Assessment of pGlu3-Aβ_42_ was performed using ELISA kit (JP27716; IBL International GmbH, Hamburg, Germany) according to the instructions of the manufacturer.

### 2.10 SDS- page and immunoblotting

Brain and liver tissues were weighted and lysed in 10 volumes of tissue homogenizing buffer (TBH; 1 M Tris, 100 mM EDTA, 40 mM EGTA containing protease and phosphatase inhibitor) using Ultra-Turrax T8 Tissue homogenizer (IKA Labor Technik, Staufen, Germany). Protein concentrations of brain and liver samples were determined using the protein bicinchoninic acid assay (Pierce™ BCA Protein Assay Kit, Thermo Fisher Scientific, Wien, Austria). 20–25 μg of protein per sample was loaded onto a 4–12% Criterion™ XT Bis-Tris Protein gel (Bio-Rad Laboratories, Wien, Austria) and electrophoresis was performed for 80 min at 140 V using MES running buffer (Bio-Rad Laboratories, Wien, Austria), to achieve proper separation of proteins. Semi-dry electrophoretic transfer of proteins to 0.20 μm nitrocellulose membranes (Bio-Rad Laboratories, Wien, Austria) was performed in a Trans-blot turbo transfer system (Bio-Rad Laboratories, Wien, Austria) at 2.5 V, 1.0 A, for 30 min. 5% non-fat dry milk (Carl Roth, Karlsruhe, Germany) in Tris-buffered saline containing 0.05% Tween 20 (TBST) was used for blocking for 1 h. Membranes were probed with primary antibodies diluted in TBST containing 5% milk powder. Membranes were further incubated in Clarity Western ECL Substrate (Bio-Rad Laboratories, Wien, Austria) and the chemiluminescent signal was detected using a ChemiDoc imager (Bio-Rad Laboratories, Wien, Austria). ImageLab software (version 5.2.1, Bio-Rad Laboratories, Wien, Austria) was used for signal quantification. All primary and secondary antibodies used are listed in Supplementary Table 3.

### 2.11 Immunofluorescent labelling and imaging

Frozen sagittally cut 10 μm thick mouse brain cryo sections were air-dried for approximately 1 h and then washed in PBS for 10 min. Additionally, sections were pretreated with ice-cold sodium borohydride/PBS solution (1 mg/ml; #213462, Sigma–Aldrich, Wien, Austria) for 4 min and washed twice in PBS for 5 min each. Following, non-specific labelling of mouse brain sections was blocked by incubating sections for 60 min with 10% donkey serum/0.1% Triton X-100/PBS. For antigen detection, sections were incubated with primary antibodies (Supplementary Table 4) in a damp chamber overnight at 4 °C. After washing, primary antibody binding was visualized by incubating sections with secondary fluorophore-conjugated antibodies (Supplementary Table 4) for 60 min. Cell nuclei were visualized by counterstaining with 40,6-Diamidin-2-phenylindol-working (DAPI) solution (#A1001; AppliChem, Monza, Italy) for 15 min and subsequently washed in PBS and ddH_2_O for 5 min each. Sections were covered with Moviol and coverslips. Imaging of immunofluorescent labelling was performed using a Zeiss AxioImager Z1 microscope (Carl Zeiss Microscopy, Oberkochen, Germany) with a high aperture lens and an AxioVision 4.8 software-driven AxioCam MRm digital camera (10x lens, numeric aperture 0.8, 1x optocoupler, Carl Zeiss Microscopy, Oberkochen, Germany). Mosaic image arrays covering the whole isocortex and hippocampus were captured at different z-levels and projected to 2D using the AxioVision 4.8 software.

### 2.12 Statistics

Basic statistical analyses were performed using GraphPad Prism 10.0 (GraphPad Prism Software, USA). Data are presented as mean ± standard error of mean (SEM) and group differences are evaluated by unpaired *t*-test, one-way ANOVA or two-way ANOVA followed by *Dunnett's or Tukey's post hoc* multiple comparisons tests.

## 3 Results

### 3.1 T2D induction impacts APPxhQC mice and their NTG littermates’ response to glucose

**Figure 1:**
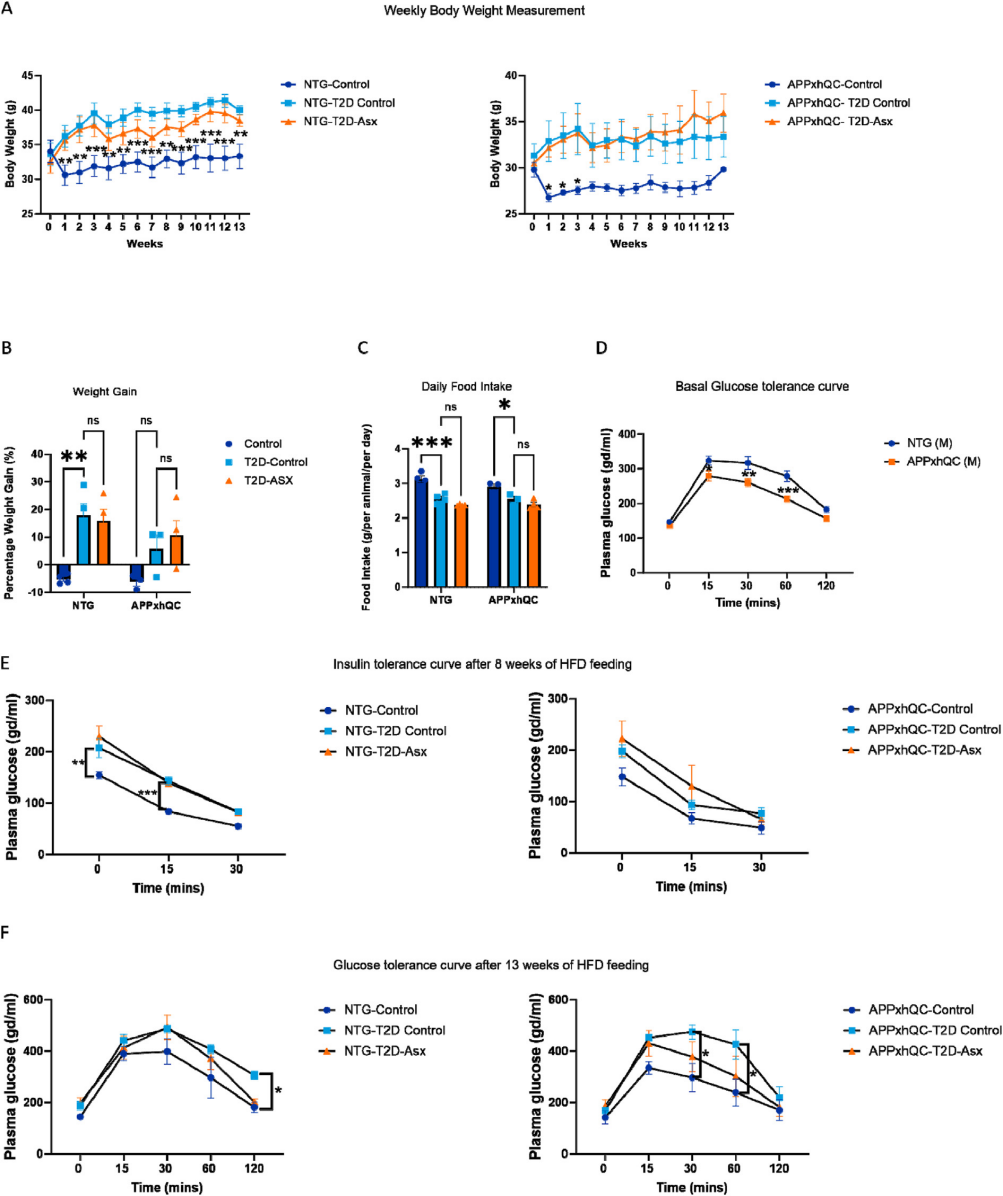
**APPxhQC mice have better glucose tolerance compared to NTG mice.** Average daily food intake of each animal and weight gain were measured weekly. Weekly weight measurement (A), percentage weight gain (B) and daily food intake (C) were evaluated over the course of 13 weeks of the study and are presented as average. Glucose tolerance test was carried out in APPxhQC and NTG mice after 5 h of fasting before the start of the T2D induction. Intraperitoneal glucose tolerance was performed at basal (D), intraperitoneal insulin tolerance in response to glucose was performed after 8 weeks of HFD feeding (E) and Intraperitoneal glucose tolerance was repeated after 13 weeks of HFD feeding (F). Data are mean ± SEM, n = 3–4 per group; *p*-values were calculated using two-way ANOVA followed by *Dunnett's* multiple comparison test. ns: not significant; ∗p < 0.05; ∗∗p < 0.01; ∗∗∗p < 0.001. Deep blue, light blue and red colours represent Control, T2D-Control and T2D-ASX groups respectively.

We evaluated the percentage of weight gain and average amount of food consumed daily during the 13 weeks of feeding. HFD feeding has been reported to reduce food intake and induce weight gain in male 3xTg-AD mice (Gali et al., 2019). A significant effect of diet on weight gain was observed (T2D induction F_2, 16_ = 12.98, p < 0.001, genotype F_1, 16_ = 3.302, p = 0.09, Figure 1B). NTG T2D-Control mice showed a significant weight gain compared to their control group (p = 0.002, Figure 1A, B). Furthermore, diet greatly influenced the amount of food consumed (F_2, 16_ = 39.58, p < 0.001, Figure 1C). Both, NTG T2D-Control (p < 0.001) and APPxhQC T2D-Control mice (p = 0.01, Figure 1C) consumed less food compared to their controls. Similarly, also the T2D-ASX group consumed less food (Figure 1C).

Prior to inducing T2D in the experimental groups, mice were subjected to basal ipGTT. At basal level, APPxhQC mice had better glucose tolerance after 15 min (p = 0.03), 30 min (p = 0.003) and 90 min (p < 0.001) of intraperitoneal glucose injection compared to NTG mice (Figure 1D). After 8 weeks of HFD feeding, mice underwent insulin tolerance test, NTG-T2D Control mice had elevated glucose at time point 0 (p = 0.003) and in response to intraperitoneal insulin injection at time point 15 (p < 0.001) compared to NTG-Control mice (Figure 1E). After 13 weeks of T2D induction, ipGTT was repeated. APPxhQC-T2D mice had elevated plasma glucose after 30 min (p = 0.02) and 60 min (p = 0.01) of intraperitoneal glucose injection compared to APPxhQC -Control mice (Figure 1F). In contrast, in the NTG group, elevated plasma glucose was seen in NTG-T2D Control mice only after 120 min (p = 0.03) of intraperitoneal glucose injection compared to NTG-T2D Control mice (Figure 1F). Taken together, these results suggest that APPxhQC mice have better glucose tolerance compared to NTG mice.

### 3.2 Glycemic parameters in T2D-induced APPxhQC and NTG mice

We evaluated the effect of APPxhQC transgenes and T2D induction on fasting plasma insulin levels (FPI), fasting plasma glucose levels (FPG), homeostatic model assessment for insulin resistance (HOMA-IR) and homeostasis model assessment of beta-cell function (HOMA-β). Although all evaluations were statistically not significant, nevertheless, T2D induced metabolic impairments were more severe in NTG mice compared to APPxhQC mice (Supplementary Table 5).

### 3.3 Plasma cholesterol but not triglyceride levels are elevated in T2D-induced APPxhQC and NTG mice

**Figure 2:**
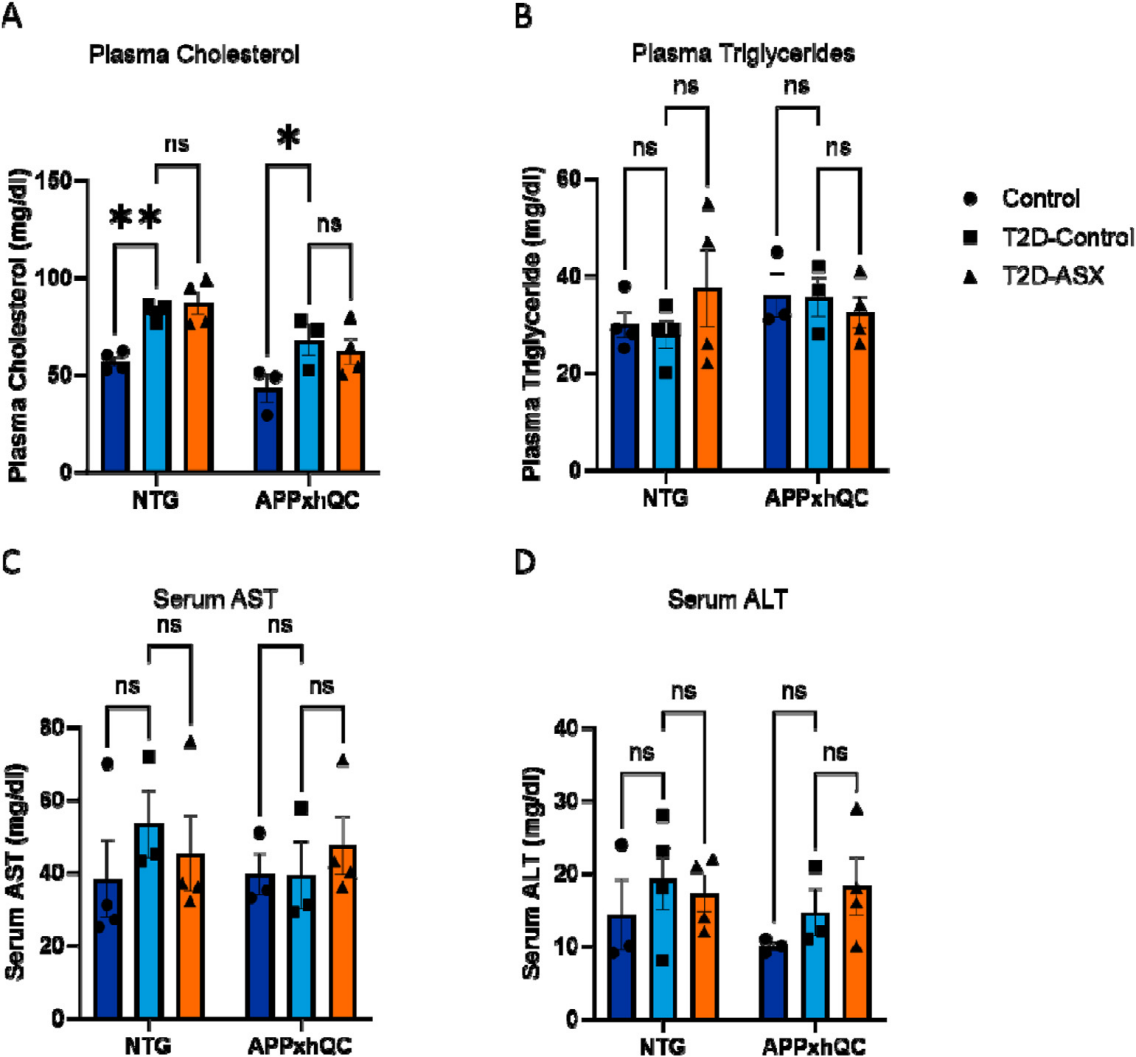
**Plasma cholesterol levels are elevated in T2D-induced APPxhQC and NTG mice.** Total plasma cholesterol levels were elevated in T2D-Control mice. Plasma cholesterol level (A); plasma triglyceride level (B); serum AST level (C) and serum ALT level (D). Data are mean ± SEM, n = 3–4 mice per group. *p*-values are calculated using two-way ANOVA followed by *Dunnett's* multiple comparison test. ns: not significant; ∗p < 0.05; ∗∗p < 0.01. Deep blue, light blue and red colours represent Control, T2D-Control and T2D-ASX groups respectively.

Dyslipidemia and impaired cholesterol metabolism are associated with both AD and T2D. We thus investigated the influence of T2D induction and pyroglutamylation on plasma cholesterol and triglyceride levels in APPxhQC and NTG mice. Diet and genotype significantly influenced cholesterol levels (T2D induction F_2, 16_ = 12.96, p < 0.001, genotype F_1, 16_ = 15.22, p = 0.001, Figure 2). T2D induction led to increased plasma cholesterol levels as both NTG T2D-Control (p = 0.009) and APPxhQC T2D-Control mice (p = 0.03) had elevated plasma cholesterol levels compared to mice in the control group (Figure 2A). Diet and genotype had no significant effect on plasma triglycerides as well as serum AST and ALT levels (Figure 2B,C and 2D, respectively). Furthermore, ASX did not influence any of these parameters.

### 3.4 T2D induction causes a shift from soluble to insoluble Aβ isoforms in the brain of APPxhQC mice

Aggregation of the Aβ protein is thought to play a central role in the pathophysiology of AD. Pathological Aβ production is proposed to be the mechanistic link that underlies the pathology of insulin resistance and diabetes in AD. To evaluate the effect of T2D induction on Aβ metabolism in APPxhQC mice, we measured the amount of soluble (DEA fractions) and plaque-associated insoluble (FA fractions) Aβ isoforms deposited in whole brain lysates of APPxhQC-Control, APPxhQC T2D-Control, and APPxhQC-T2D-ASX mice.

**Figure 3:**
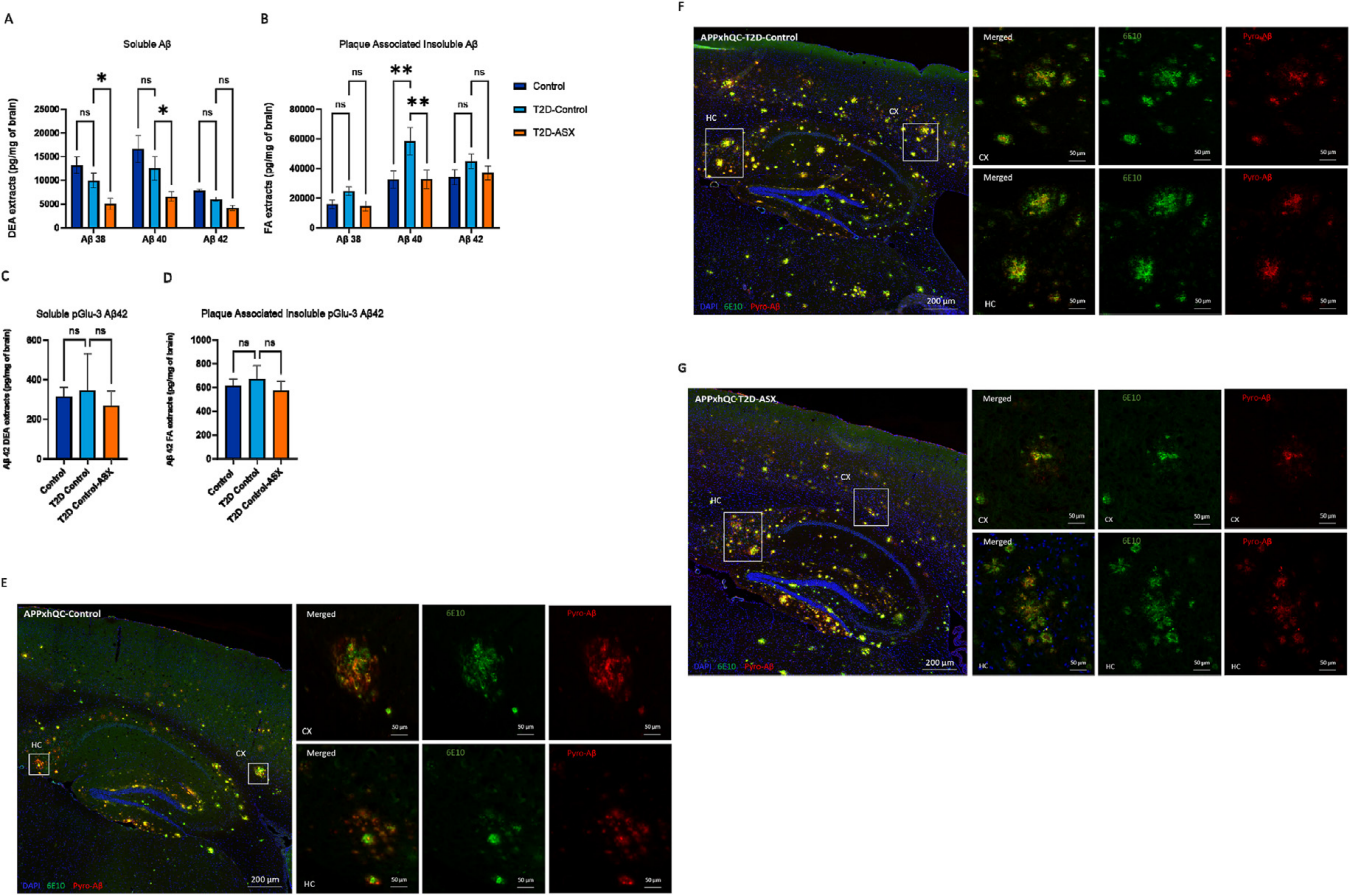
**ASX treatment reduces Aβ levels in T2D-induced APPxhQC male mice.** Levels of soluble and plaque-associated insoluble Aβ isoforms in the brain of APPxhQC mice were evaluated by MSD assay. DEA-extracted soluble Aβ_38_, Aβ_40_ and Aβ_42_ levels (A); FA-extracted plaque-associated insoluble Aβ_38_, Aβ_40_ and Aβ_42_ levels (B). DEA-extracted soluble pGlu-3 Aβ_42_ (C); FA-extracted plaque-associated insoluble pGlu-3 Aβ_42_ (D); Data are mean ± SEM, n = 3–4 mice per group. *p*-values are calculated using two-way ANOVA for A and B and one-way ANOVA for C and D followed by *Dunnett's* multiple comparison *post hoc* test. ns: not significant; ∗p < 0.05; ∗∗p < 0.01. (E-G) Representative micrographs of immunofluorescence labeling for Aβ (6E10); pyroglutamate modified Aβ Glu-3 Aβ_42_, and nuclei (DAPI) in APPxhQC Control (E), APPxhQC T2D-Control (F) and APPxhQC T2D-ASX (G). HC, hippocampus; CX, cortex. Labelings for 6E10 Aβ are in green, pGlu-3 Aβ in red and DAPI in blue. Areas depicted by the rectangle in the left overview images are shown enlarged in the images on the right. Deep blue, light blue and red colours represent Control, T2D-Control and T2D-ASX groups respectively.

We observed that soluble Aβ isoforms were significantly affected by T2D induction and genotype (F_2, 21_ = 18.86, p < 0.001, F_2, 21_ = 7.180, p = 0.004, respectively; Figure 3A). Although, T2D induction did not change the concentration of soluble Aβ_38_ and Aβ_40_ deposits compared to the control group, ASX treatment led to a significant reduction in the deposited soluble Aβ_38_ and Aβ_40,_ levels compared to the T2D-Control group (p = 0.05, p = 0.02 respectively, Figure 3A). Furthermore, T2D induction led to a significant increase in the amount of plaque-associated insoluble Aβ_40_ deposits compared to the control group (p = 0.007, Figure 3B), while ASX supplementation significantly reduced the amount of plaque-associated insoluble Aβ_40_ deposits (p = 0.005, Figure 3B). No differences in soluble or plaque-associated insoluble Aβ_42_ in APPxhQ-T2D-Control mice were observed (Figure 3A,B, respectively).

In addition, we evaluated the impact of T2D induction on pyroglutamylation by assessing the amounts of soluble and plaque-associated insoluble pGlu-3 Aβ_42_ deposits by ELISA. No differences between groups were observed (Figure 3C,D).

In addition, immunolabeling for 6E10 and pGlu-Aβ in hippocampal and cortical sections of the brain of APPxhQC mice were performed. Both 6E10 and pGlu-3 Aβ were localized in the same clusters. We observed that 6E10-positive plaques appeared spread out, while that of pGlu-3 Aβ were visible in narrow spots. T2D induction did not change the immunoreactive area and object density of 6E10 and pGlu-3 Aβ in the hippocampus and cortex relative to the control group (Figure 3E–G; Supplementary Figures 1). ASX treatment did also not influence these parameters (Figure 3E–G; Supplementary Figures 1).

### 3.5 ASX treatment rescues T2D-induced hippocampus-dependent cognitive impairment in NTG mice

To investigate if ASX treatment can improve spatial learning, all mice were subjected to hippocampus-dependent behavioral testing. The Morris water maze (MWM) was used to evaluate spatial learning and memory performance.

**Figure 4:**
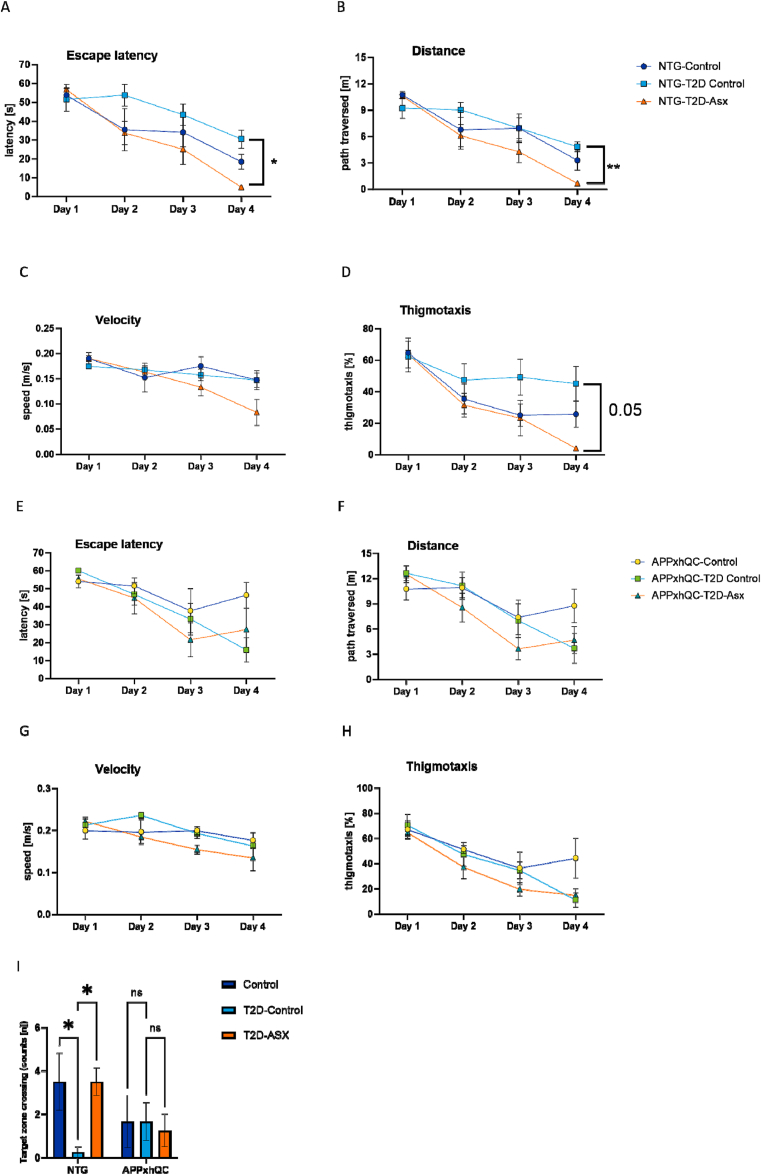
**ASX treatment rescues T2D-induced spatial deficit in T2D-induced NTG mice.** Escape latency, distance moved and thigmotaxis was significantly improved in ASX-treated NTG T2D-Control mice during the four-days training phase of the MWM. Escape latency (A); length of path to find hidden platform (distance; B); velocity to find hidden platform (C) and thigmotaxis (D) in NTG mice. Escape latency (E); length of path to find hidden platform (distance; F); velocity to find hidden platform (G) and thigmotaxis (H) in APPxhQC mice. Number of target zone crossings made by both APPxhQC and NTG mice on day 5 of MWM during the probe trial (I). Data are mean ± SEM, n = 3–4 mice per group. *p*-values were calculated using two-way ANOVA followed by *Dunnett's* multiple comparison *post hoc* test. ns: not significant; ∗p < 0.05; ∗∗p < 0.01. Deep blue, light blue and red colours represent Control, T2D-Control and T2D-ASX groups respectively.

In the training phase of the MWM test, NTG T2D-ASX mice needed less time to find the hidden platform as shown by a reduced escape latency (p = 0.02, Figure 4A) and a decreased length of path to find the hidden platform (p = 0.005, Figure 4B) after 4 days of training compared to controls. NTG T2D-ASX mice swam tentatively slower (p = 0.76, Figure 4C). Thigmotaxis, an index of anxiety, was not significantly reduced in NTG T2D-ASX mice (p = 0.05, Figure 4D).

For the probe trial, the hidden platform was removed, and the number of target zone crossings and time spent in target quadrant (SW) were evaluated. NTG T2D-Control mice made lesser target zone crossings compared to the control group (p = 0.04, Figure 4I), suggesting spatial memory deficits in this group. ASX treatment reversed this memory deficit as NTG T2D-ASX mice made more target quadrant crossings compared to the NTG T2D-control group (p = 0.04, Figure 4I).

In contrast to NTG mice, ASX treatment did not improve hippocampus-dependent cognition in APPxhQC mice (Figure 4E,F, G, H, I).

### 3.6 T2D induction and ASX treatment influence expression levels of various hepatic nutrient sensing markers in NTG mice

To unravel the effect of T2D induction and genotype on hepatic nutrient sensing pathways such as insulin, autophagy and inflammation signaling, we evaluated protein levels of several hepatic proteins in the liver.

**Figure 5:**
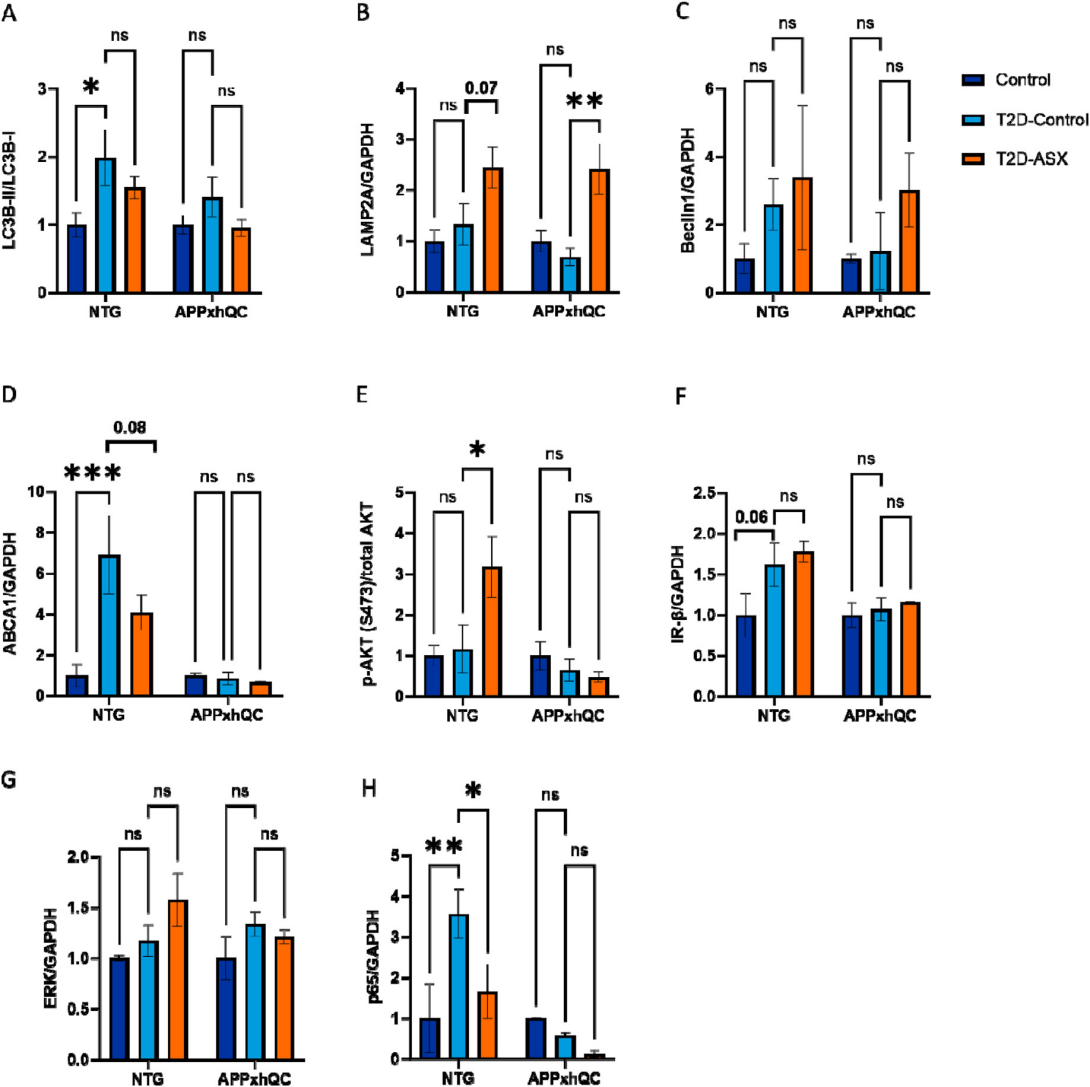
**T2D induction and ASX treatment influence expression levels of different hepatic nutrient sensing makers in NTG mice.** Quantitative analysis of immunoblots for protein markers of autophagy (LC3B, LAMP2A and Beclin1), insulin signaling (ABCA1, AKT and IR-β), and inflammation (ERK and p65) in the liver of APPxhQC and NTG mice. Densitometric analysis of LC3B (A), LAMP2A (B), Beclin1 (C), ABCA1 (D), AKT (E), IR-β (F), ERK (G), and p65 (H) expression normalized to GAPDH. Data are mean ± SEM, n = 3 mice per group. *p*-values are calculated using two-way ANOVA followed by *Dunnett's* multiple comparison *post hoc* test. ns: not significant; ∗p < 0.05; ∗∗p < 0.01; ∗∗∗p < 0.001. Deep blue, light blue and red colours represent Control, T2D-Control and T2D-ASX groups respectively.

First, we investigated the influence of T2D induction and genotype on hepatic autophagy signaling. T2D induction (F_2, 12_ = 4.305, p = 0.04) and genotype (F_1, 12_ = 3.939, p = 0.07) influenced lipidation of hepatic LC3B. We observed a significant increase in LC3B-II/LC3B-1 expression in NTG T2D-Control but not APPxhQC T2D-Control mice compared to their control groups (p = 0.02, Figure 5A, Supplementary Figure 2A). A significant effect of T2D induction on autophagic protein markers LAMP2A and Beclin1 expressions was observed (F_2, 12_ = 11.66, p = 0.002 and F_2, 12_ = 5.664, p = 0.02, Figure 5B, C, Supplementary Figure 2A respectively). ASX treatment did not significantly change the expression of LAMP2A in NTG (p = 0.07, Figure 5B, Supplementary Figure 2A) but increased LAMPS2A expression in APPxhQC mice (p = 0.007, Figure 5B, Supplementary Figure 2A) compared to the corresponding T2D-induced mice.

To investigate hepatic insulin signaling, we investigated the effects of both diet and genotype on protein expressions of ABCA1, AKT and IR-β. Both, T2D induction (F_2, 12_ = 5.259, p = 0.02) and genotype (F_1, 12_ = 18.93, p < 0.001), significantly influenced ABCA1 expression in NTG but not APPxhQC mice. The genotype had a significant effect on phosphorylation of AKT (F_2, 12_ = 8.615, p = 0.01) and IR-β protein expression (F_1, 12_ = 6.702, p = 0.02). Notably, ABCA1 protein expression was highly upregulated in NTG T2D-Control mice compared to the control group (p = 0.001, Figure 5D, Supplementary Figure 2A). ASX treatment could sightly reduce ABCA1 levels but data were not significant compared to the NTG T2D-Control group (p = 0.36, Figure 5D, Supplementary Figure 1B). In livers of NTG T2D-ASX mice, we observed an increased expression of phosphorylated AKT compared to the NTG T2D-Control group (p = 0.01, Figure 5E, Supplementary Figure 2B). NTG T2D-Control mice further present slightly but not significantly increased IR-β protein levels compared to the control group (p = 0.06, Figure 5F, Supplementary Figure 2B). In addition, we checked the effects of T2D induction and genotype on protein expressions of hepatic ERK and p65. We observed a significant effect of T2D induction on the expressions of p65 (F_2, 12_ = 4.956, p = 0.03, Figure 5H, Supplementary Figure 2C) and a trend for ERK (F_2, 12_ = 2.956, p = 0.09, Figure 5G, Supplementary Figure 2C). Moreover, a strong association was observed for T2D induction and genotype for p65 protein expression (F_2, 12_ = 7.662, p = 0.007). In addition, we observed an increased hepatic protein expression of p65 in NTG T2D-Control mice compared to the control group (p = 0.007, Figure 5H, Supplementary Figure 2C). ASX treatment significantly reduced the expression of p65 (p = 0.04, Figure 5H, Supplementary Figure 2C).

In contrast to NTG mice, all hepatic parameters investigated were not changed in a statistically significant way in APPxhQC mice. Only LAMP2 was increased in APPxhQC T2D-ASX mice compared to controls (Figure 5A–H).

Taken together, these data show that T2D induction increases several hepatic markers in NTG but not APPxhQC mice. ASX treatment was able to slightly reverse this effect in NTG mice, while increasing expression levels of markers that were unchanged by T2D induction. Changes observed in APPxhQC mice were overall much weaker.

### 3.7 ASX treatment increases cerebral mTORC1 expression in NTG T2D-Control mice

**Figure 6:**
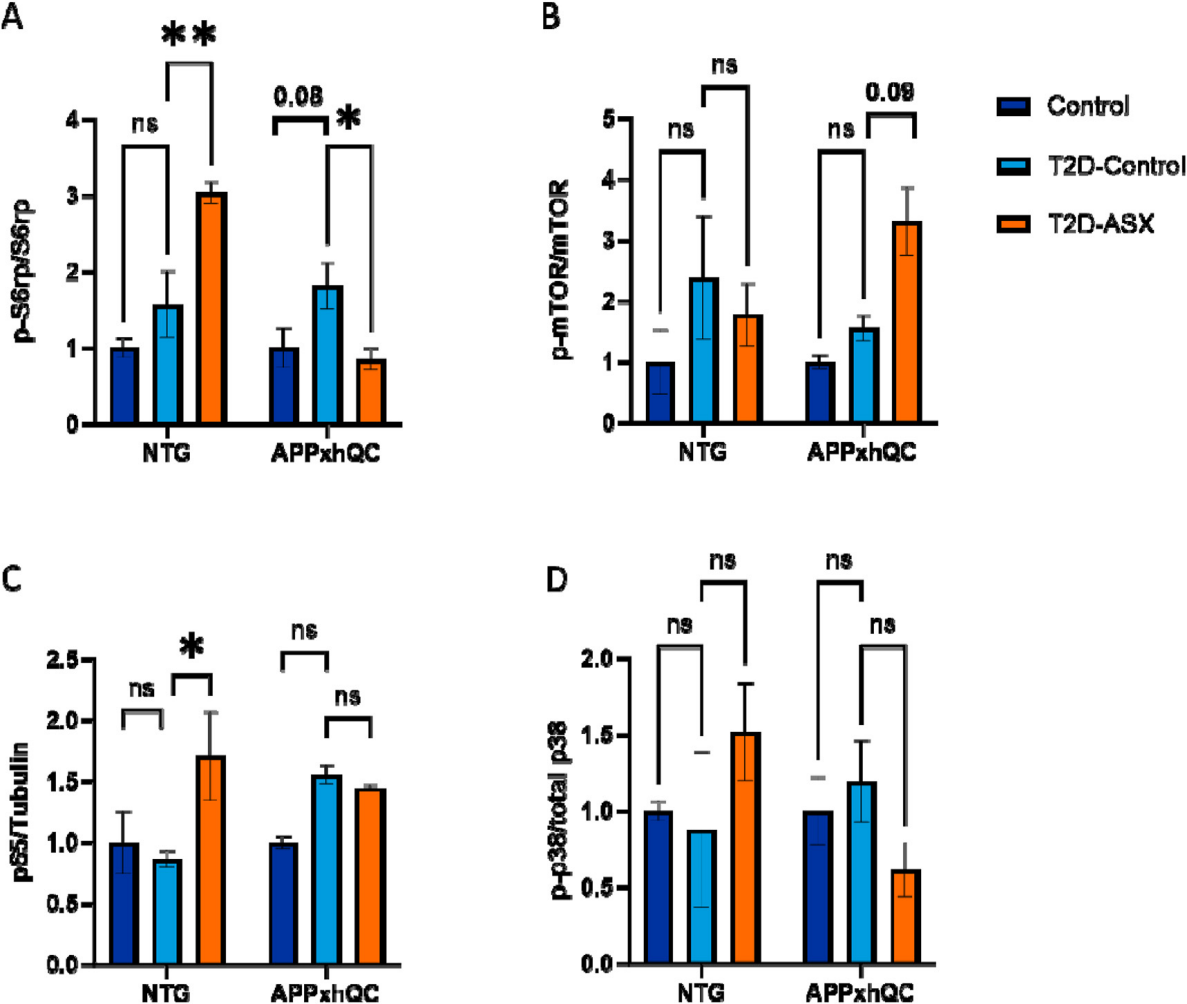
**ASX treatment increases cerebral mTORC1 andMAPKinase *signalling in NTG T2D-Control mice.*** Quantitative analysis of immunoblots for mTORC1 (S6rp and mTOR) and inflammatory protein markers (p65 and p38) in the brain of NTG and APPxhQC mice. Densitometric analysis of S6rp (A), mTOR (B), p65 (C), and p38 (D) expressions normalized to tubulin. Data are mean ± SEM, n = 3 mice per group. *p*-values are calculated using two-way ANOVA followed by *Dunnett's* multiple comparison *post hoc* test. ns: not significant; ∗p < 0.05; ∗∗p < 0.01. Deep blue, light blue and red colours represent Control, T2D-Control and T2D-ASX groups respectively.

Phosphorylation of the S6 ribosomal protein (S6rp) is a common readout for the activation of the mammalian target of rapamycin complex 1 (mTORC1) and marker for neuronal activity [[85], [86], [87], [88]]. We evaluated the effects of T2D induction and genotype on the phosphorylation of S6rp in the brain. T2D induction significantly influenced phosphorylation of S6rp (F_2, 12_ = 6.957, p = 0.01, Figure 6A, Supplementary Figure 3A). There was a strong interaction between T2D induction and genotype (F_2, 12_ = 13.07, p < 0.001). NTG T2D-ASX mice showed increased phosphorylation of S6rp compared to the NTG T2D-Control group, while a down-regulation was observed in APPxhQC T2D-ASX mice (p = 0.004, p = 0.04, respectively; Figure 6A, Supplementary Figure 3A). Also, we evaluated the effect of T2D induction and genotype on the phosphorylation of mammalian target of rapamycin (mTOR) protein. T2D induction did not significantly increase phosphorylation of mTOR (F_2, 12_ = 3.743, p = 0.05, Figure 6B, Supplementary Figure 3A). Furthermore, we investigated the impact of T2D induction and the APPxhQC genotype on brain expression of p38 and p65 NF*-κB, both* members of the MAPK pathway and implicated in adult hippocampal neurogenesis (AHN). T2D induction had a significant impact on p65 expression (F_2, 12_ = 4.878, p = 0.03, Figure 6C, Supplementary Figure 3B). NTG T2D-ASX mice presented an increased expression of p65 compared to the control group (p = 0.01, Figure 6C, Supplementary Figure 3B). Phosphorylation of p38 protein was not changed by T2D induction or genotype (p = 0.25 and p = 0.32, respectively, Figure 6D, Supplementary Figure 3B).

## 4 Discussion

### 4.1 Discussion

In this study we investigated combinatory effects of T2D induction and the APPxhQC transgenes on glucose sensitivity, spatial learning, and nutrient sensing signalling. We observed that T2D induction led to reduced food consumption in NTG as well as APPxhQC mice but massive weight gain only in NTG. This agrees with similar reports by Sankar et al. [89] and Mazzei and colleagues [90] who reported weight gain in NTG mice but not in APP/PSI and App^NL−F/NL−F^ knock-in mice, respectively. APPxhQC transgenic mice did not significantly gain weight.

### 4.2 Effects of T2D

Although induction of T2D caused only a slight reduction in glucose sensitivity in APPxhQC mice, it caused a significant increase of plasma cholesterol levels in NTG and APPxhQC mice. This partially agrees with a study published by Skrzypski and colleagues where they reported that T2D induction by HFD-streptozocin treatment leads to elevated levels of triacylglycerol and cholesterol [91]. In vitro studies as well as *in vivo* studies in Apo E deficient and C57BL/6J mice have shown that elevated extracellular and intracellular cholesterol levels lead to functional disturbances in pancreatic beta cells [[92], [93], [94]]. In addition, evidence suggests that hypercholesterolemia itself could reduce glucose-stimulated insulin secretion and insulin sensitivity [92,95], which agrees with the reduced ability to secrete insulin as observed in NTG T2D-Control mice in our study.

Induction of T2D further impaired the memory in NTG mice. Cognitive impairment due to T2D induction in rats [96,97] and mice [98,99] is well reported. Induction of T2D further impaired the memory in NTG mice but not in APPxhQC mice. Variations in the impact of T2D on cognitive function in this study are not outright surprising as reports about the effect of T2D on cognitive performance in experimental animal models have been varying; ranging from increased Aβ deposition due to T2D induction accompanied by memory impairments [90,[100], [101], [102], [103], [104]] to no memory impairment or only mild memory dysfunction [[105], [106], [107]]. Some studies even reported impaired memory function due to T2D induction without increased Aβ deposition [108,109].

Induction of T2D further upregulated the hepatic protein levels of LC3B and ABCA1 in NTG mice. Increased level of hepatic LC3B might have resulted from defective autophagy caused by nutrient overload leading to the accumulation of LC3B. Similarly, increased levels of hepatic ABCA1 might have been due to cholesterol overload as we observed increased total plasma cholesterol in these mice. In addition, T2D induction can cause an increase of some Aβ isoforms, which might lead to an increase in ABCA1 levels. In agreement with this, Azizidoost and colleagues [110] showed that Aβ can cause a significant increase in ABCA1 protein levels. Furthermore, Liang et al. [111] reported that activation of autophagy leads to an increased expression of ABCA1. Increased expression of these two proteins - ABCA1 and LC3B - in NTG T2D-Control mice are likely a result of the effect of T2D induction on endogenous Aβ metabolism.

The effect of T2D induction was much weaker in APPxhQC mice, mainly causing a shift from the soluble to insoluble Aβ pool and an increase of insoluble Aβ40 levels. This shift from the soluble to insoluble Aβ pool is similar to results by Wang et al. [112]. Their findings in *post-mortem* brain of AD patients showed that the onset and progression of AD are significantly influenced by the conversion of soluble forms of Aβ_40_ and Aβ_42_ to insoluble forms accompanied by significantly increased levels of insoluble Aβ_40_ [112]. In contrast, in studies using APP/PS1xdb/db mice, increased expression of soluble Aβ_40_ and Aβ_42_ and reduced deposition of insoluble Aβ_40_ and Aβ_42_ in the cortex and hippocampus were reported after HFD, connoting a shift in Aβ levels towards more toxic soluble species [89,113,114]. Conversely, Carus-Cadavieco and colleagues [104] reported a reduction in both soluble and insoluble Aβ in streptozocin-HFD-fed hAPP NL/F mice. HFD increased the deposition of insoluble Aβ in hAPP NL/F but not of soluble Aβ [90]. In 3xTg-AD and Tg 2576 mice, HFD caused an increase in soluble Aβ_40_ and Aβ_42_ [103,115,116] but no change was observed in AppNL/NL mice [107].

These findings in combination with the observed shift from soluble to insoluble Aβ isoforms in the brain of APPxhQC T2D-Control mice used in this study suggest that peripheral metabolic dysfunctions, such as glucose intolerance caused by HFD and streptozocin, affect Aβ metabolism - deposition, clearance, and degradation.

### 4.3 Ameliorating effect of ASX treatment

Treatment of T2D-induced mice with ASX only slightly improved glucose sensitivity in NTG and APPxhQC mice, but it reversed learning and memory deficits, increased levels of hepatic markers IR-β and p-AKT while reducing others, and increased p-S6rp and p65 levels in the brain of NTG mice. The increased expression of hepatic IR-β and p-AKT levels in NTG T2D-Asx mice suggests an improved insulin signalling as these proteins are needed for glucose homeostasis. Yook and colleagues reported that ASX treatment increases AHN, plasticity, and spatial memory in male C57BL/6J mice fed diet supplemented with ASX [117]. Moreover, *AHN is well known to have a positive effect on behavioral learning and memory tasks* [118]*. In addition, increased expression of* p-S6rp in the brain might suggests the selective activation of mTOR signaling. This selective activation of mTOR might be responsible for improved cognitive function in these mice as mTOR signaling is necessary for memory formation and acute inhibition of mTORC1 may greatly impact synaptic function and, consequently, impair memory retrieval [[119], [120], [121]]. p65 expression has been linked with AHN [[122], [123], [124], [125]]. The increased expression of brain p65 and selective activation of mTOR point in the same direction of improved AHN.

ASX treatment reversed impaired hippocampal dependent spatial memory function in NTG T2D-Control mice in our study. These mice spent less time to find the hidden platform, swam a shorter distance, swam slower and showed reduced thigmotaxis, an immediate response to anxiety during the training phase. Furthermore, we recorded more target zone crossings during the probe trial in NTG T2D-ASX mice suggesting a potential of ASX treatment to reverse spatial memory deficits caused by T2D.

ASX's effect on Aβ_40_ expression (soluble and insoluble) in APPxhQC mice in this study, agrees with a recent publication from our group where we reported that co-treatment of ASX with Aβ_40_ enhances Aβ clearance in primary porcine brain capillary endothelial cells (pBCECs) by reducing intracellular and secreted APP/Aβ *in vitro* [77].

In this study, ASX treatment might have rescued spatial deficits in T2D-induced NTG mice probably by improving glucose metabolism, mTOR-p65 and brain-liver nutrient sensing signaling.

### 4.4 Probable protective effect of the APPxhQC transgene in the context of T2D comorbidity and AD

We observed less differences caused by T2D in APPxhQC mice than in NTG mice. This might be a consequence of one of the transgenes, probably hQC, which is protective in the context of T2D pathology. Even though ASX treatment reduced Aβ expressions in APPxhQC T2D-Control mice, it caused no cognitive improvement in these mice. This could either be because treatment was introduced in combination with another risk factor- T2D, when AD pathology was already in advanced stage or due to the fact that disease pathophysiology in context of pGlu Aβ-induced AD pathology and T2D appears different. Pyroglutamylation (hQC) down-regulates the expression of neprilysin (NEP), while T2D upregulates it. Huang et al. [126] suggested that when neprilysin activity is lost, N-terminal truncation of Aβ and conversion of pGlu Aβ is possible. In humans, NEP levels inversely correlate with Aβ deposition in both demented and non-demented individuals [127,128] Upregulation of NEP is thus one way to facilitate Aβ degradation. Increased expression of NEP was reported in plasma and metabolic tissues such as liver, kidney, epididymal, mesenteric and perirenal fat of mice with diet-induced obesity, its expression level correlated with decreased insulin sensitivity and reduced beta cell function [129,130]. Further studies are needed to clarify the protective effect of the APPxhQC transgene in the context of T2D comorbidity and AD.

## 5 Summary

In conclusion, our study confirms that T2D poses an additional risk factor for AD development and suggests, that it could result in metabolic dysfunction-induced cognitive impairments. ASX treatment reduces Aβ expressions and rescues hippocampus-dependent memory impairment.

## Funding

This work was funded by the Austrian Science Fund (10.13039/501100002428FWF) in the doctoral program *Metabolic and Cardiovascular Disease* (DK-MCD W1226) at the 10.13039/501100010109Medical University of Graz with support from QPS Austria GmbH.

## CRediT authorship contribution statement

**Joshua Adekunle Babalola:** Writing – review & editing, Writing – original draft, Project administration, Methodology, Investigation, Formal analysis, Data curation, Conceptualization. **Anika Stracke:** Writing – review & editing, Resources, Data curation. **Tina Loeffler:** Writing – review & editing, Supervision, Methodology. **Irene Schilcher:** Writing – review & editing, Supervision, Methodology. **Sideromenos Spyridon:** Writing – review & editing, Supervision, Methodology, Formal analysis, Data curation. **Stefanie Flunkert:** Writing – review & editing, Supervision. **Joerg Neddens:** Writing – review & editing, Supervision, Data curation. **Ake Lignell:** Writing – review & editing, Resources, Methodology. **Manuela Prokesch:** Writing – review & editing, Supervision, Resources. **Ute Pazenboeck:** Funding acquisition, Conceptualization. **Herbert Strobl:** Writing – review & editing, Resources, Funding acquisition. **Jelena Tadic:** Writing – review & editing, Supervision, Formal analysis. **Gerd Leitinger:** Writing – review & editing, Supervision, Formal analysis. **Achim Lass:** Writing – review & editing, Supervision, Formal analysis. **Birgit Hutter-Paier:** Writing – review & editing, Supervision, Resources, Funding acquisition. **Gerald Hoefler:** Writing – review & editing, Supervision, Funding acquisition.

## Declaration of competing interest

T.L., I.S., S.S., S.F., J.N., M.P. and B.H.P. are presently or formerly affiliated with QPS Austria GmbH. S.F. is further affiliated with BioDoks e. U.

## Data Availability

Data will be made available on request.
