# Corrigendum to “Effect of astaxanthin in type-2 diabetes -induced APPxhQC transgenic and NTG mice” [MOLMET 85 (2024) 1–16]

**DOI:** 10.1016/j.molmet.2024.101972

**Published:** 2024-06-23

**Authors:** Joshua Adekunle Babalola, Anika Stracke, Tina Loeffler, Irene Schilcher, Spyridon Sideromenos, Stefanie Flunkert, Joerg Neddens, Ake Lignell, Manuela Prokesch, Ute Panzenboeck, Herbert Strobl, Jelena Tadic, Gerd Leitinger, Achim Lass, Birgit Hutter-Paier, Gerald Hoefler

**Affiliations:** 1 D&F Institut für Pathologie Medizinische Universität Graz, Graz, Austria; 2 Division of Immunology and Pathophysiology, Otto Loewi Research Center, Medical University of Graz, Austria; 3 QPS Austria GmbH, Grambach, Austria; 4 Medical University of Vienna, Vienna, Austria; 5 AstaReal AB, Sweden; 6 Division of Cell Biology, Histology and Embryology, Gottfried Schatz Research Center, Medical University of Graz, Austria; 7 Institute of Molecular Biosciences, University of Graz, Austria

We regret the misspelling of the name of ‘**Ute Panzenboeck**’ one of the authors written as Ute Pazenboeck instead of **Ute Panzenboeck**. We would like to apologize for any inconvenience caused.

