# Supplementary material for: Effect of astaxanthin in type-2 diabetes -induced APPxhQC transgenic and NTG mice
Source: Mol Metab. 2024 May 17;85:101959. doi: 10.1016/j.molmet.2024.101959 (PMC11153249; doi:10.1016/j.molmet.2024.101959)
Supplement: Multimedia component 1 [file mmc1.doc]

**Supplementary Table 1: Composition of control diet 10 kJ% fat, 7% sucrose (Lard/Soybean** oil) (EF D12450J, Ssniff® diets

| **Crude Nutrient** | **%** |
| --- | --- |
| Crude Protein | 18.2 |
| Crude Fat | 4.1 |
| Crude Fibre | 5.0 |
| Crude Ash | 5.3 |
| Starch | 43.5 |
| Sugar | 6.8 |
| **Additives** | **Quantity** |
| Vitamin A | 15,000 (IE/IU) |
| Vitamin D3 | 1,500 (IE/IU) |
| Vitamin E | 150 mg |
| Vitamin C | 30 mg |
| Copper | 12 mg |

**Supplementary Table 2: Composition of HFD** diet – EF D12492 (95%) + 5% Maltodextrin Placebo (EF D12492, Ssniff® diets)

| **Crude Nutrient** | **%** |
| --- | --- |
| Crude Protein | 23.2 |
| Crude Fat | 33.1 |
| Crude Fibre | 5.7 |
| Crude Ash | 5.2 |
| Starch | 0.1 |
| Sugar | 9.0 |
| **Additives** | **Quantity** |
| Vitamin A | 14,250 (IE/IU) |
| Vitamin D3 | 1,425 (IE/IU) |
| Vitamin E | 144 mg |
| Vitamin C | 29 mg |
| Copper | 13 mg |

**Supplementary Table 3**: List of antibodies used for western blotting

| Antibody | Dilution | Company | Order No |
| --- | --- | --- | --- |
| Mouse anti-ABCA1 | 1:1,000 | Abcam | AB_18180 |
| Rabbit-anti-LC3B | 1:1,000 | Cell Signaling Technology Leiden, The Netherlands | 2775S |
| Rabbit-anti-S6rp (5G10) #2217 | 1:1,000 | Cell Signaling Technology Leiden, The Netherlands | 2217T |
| Rabbit-anti-p-S6rp (2F9) (Ser235/236) (2F9) #4856 | 1:1,000 | Cell Signaling Technology Leiden, The Netherlands | 4856S |
| Rabbit-anti-p-mTOR (D9C2) XP  (Ser2448) (D9C2) XP #5536 | 1:1,000 | Cell Signaling Technology Leiden, The Netherlands | 5536T |
| Mouse-anti-mTOR (L27D4) #4517 | 1:1,000 | Cell Signaling Technology Leiden, The Netherlands | 4517S |
| Mouse-anti-Tubulin (DM1A) #3873 | 1:1,000 | Cell Signaling Technology | 3873S |
| Goat anti-rabbit IgG-HRP | 1:10,000 | Cell Signaling Technology Leiden, The Netherlands | 7074S |
| Goat anti-mouse IgG-HRP #7076S | 1:5,000 | Cell Signaling Technology | 7076S |
| Rabbit -anti-AKT (pan) (11E7) #4685 | 1:2,000 | Cell Signaling Technology Leiden, The Netherlands | 4685S |
| Rabbit-anti-p-AKT (Ser473) (D9E) XP #4060 | 1:2,000 | Cell Signaling Technology Leiden, The Netherlands | 4060S |
| Rabbit-anti- p44/42 MAPK (Erk 1/2) (137FS) # 4695S | 1:2,000 | Cell Signaling Technology Leiden, The Netherlands | 4695S |
| Rabbit-anti-p-p44/42 MAPK (T202/ Y204) #9101S | 1:2,000 | Cell Signaling Technology | 9101S |
| Rabbit-anti-p-65NFκB (D14E12) XP #8242 | 1:2,000 | Cell Signaling Technology Leiden, The Netherlands | 8242S |
| Rabbit-anti-p38-MAPK | 1:2,000 | Cell Signaling Technology | 9212S |
| Rabbit-anti-p-p38-MAPK (Thr180/Tyr182) (D3F9) XP #4511 | 1:2,000 | Cell Signaling Technology Leiden, The Netherlands | 9211S |
| Rabbit-anti-IR-β (4B8) | 1:1,000 | Cell Signaling Technology Leiden, The Netherlands | 3025T |
| Rabbit-anti-LAMP2A | 1:1,000 | Abcam | AB_18528 |
| Rabbit-anti-Beclin1 (D40C5) #3495 | 1:1,000 | Cell Signaling Technology Leiden, The Netherlands | 3495S |
| Rabbit-anti-ATG5 (D5F5U) #12994T | 1:1,000 | Cell Signaling Technology Leiden, The Netherlands | 12994T |

**Supplementary Table 4: List of antibodies used for immunofluorescent labelling** (IF)

| Antibody | Dilution | Company | Order No |
| --- | --- | --- | --- |
| Mouse monoclonal [6E10] anti-amyloid beta, conjugated Alexa Fluor 488 | 1:2,000 | BioLegend | B803013 |
| Rabbit Abeta-pE3 antibody | 1:2,000  1:2000 | Synaptic Systems G | AB_2056424 |
| Donkey Anti-Rabbit IgG  H&L DyLight 650 | 1:500 | Abcam | AB_96922 |
| DAPI |  | Appli Chem | A1001.0025 |

**Supplementary Table 5: Glycemic Parameters**


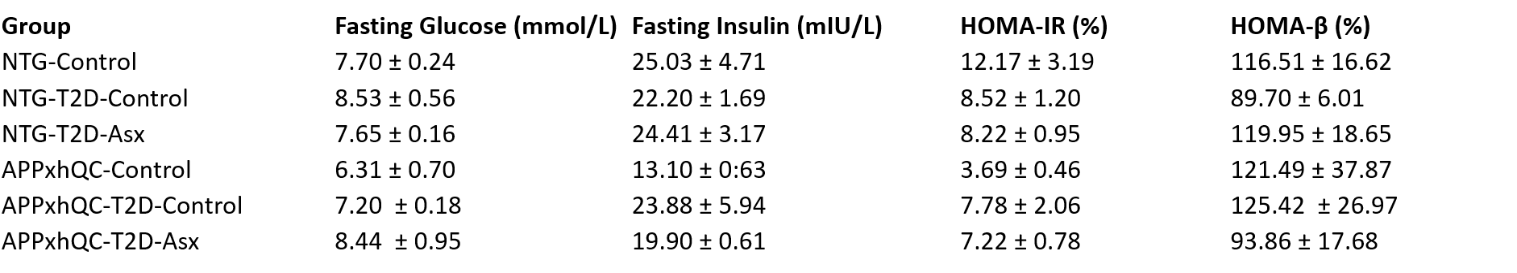


Fasting glucose, fasting insulin, HOMA-IR and HOMA-β were evaluated after 5 hours of fasting at the terminal end of the study, prior to tissue sampling. Data are mean ± SEM, n=3-4 mice per group.


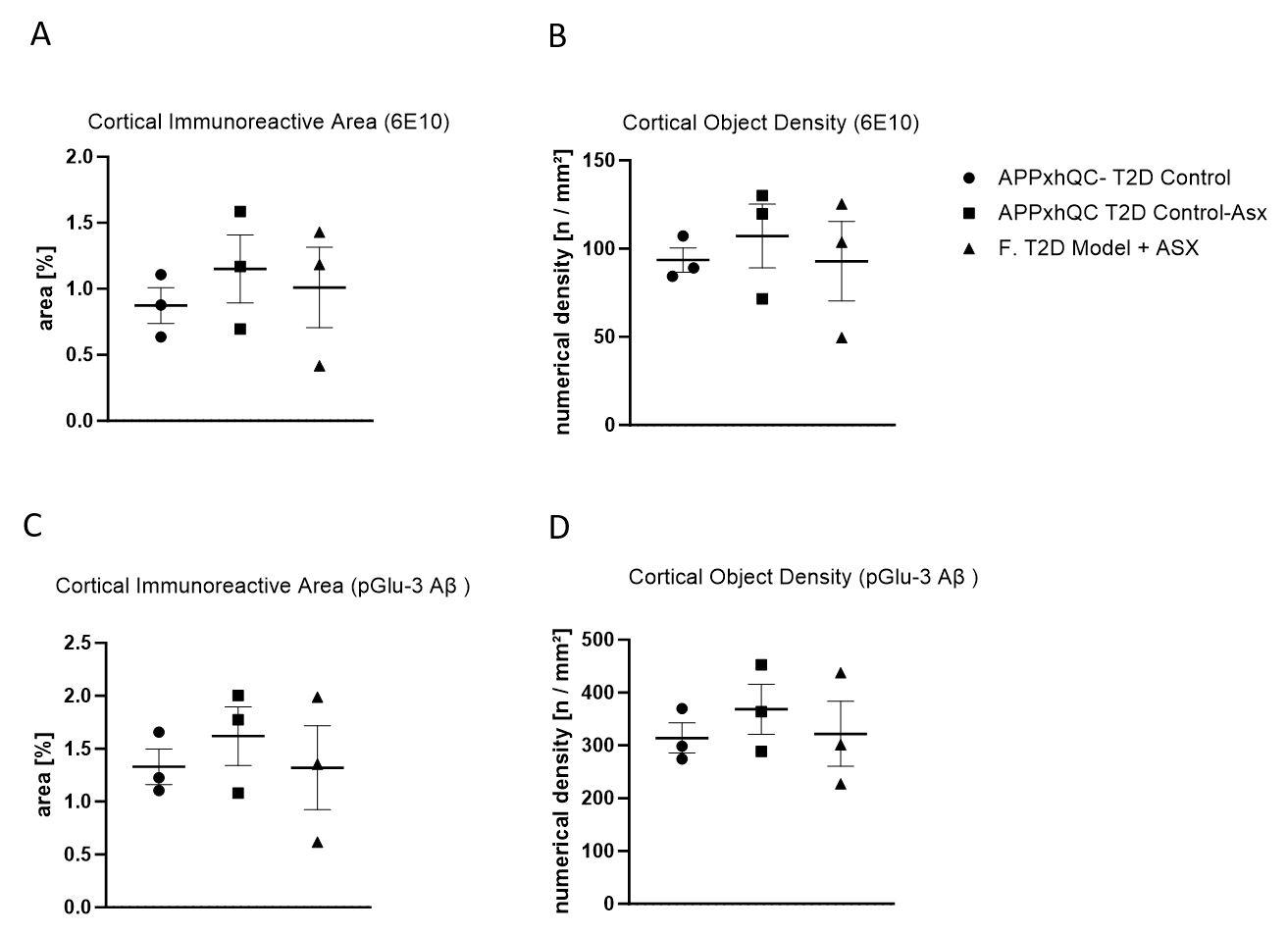


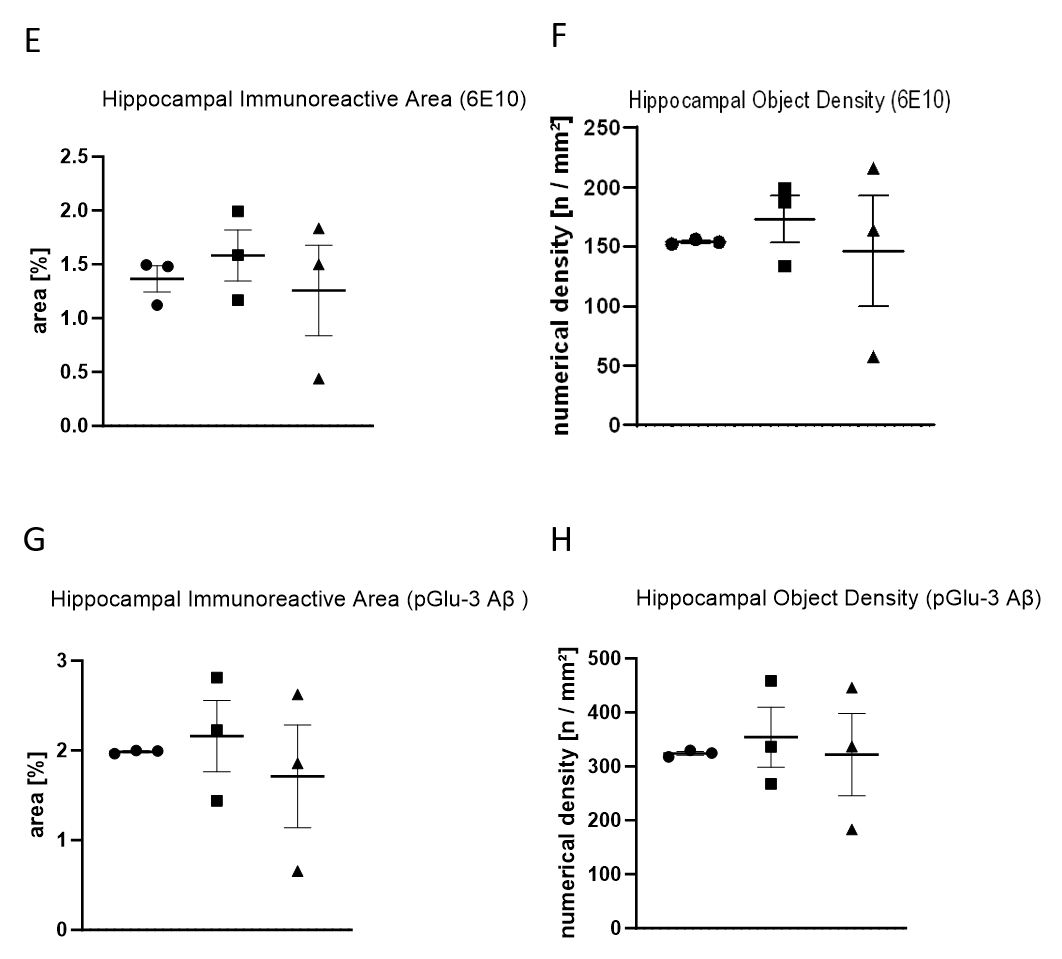


**Supplementary Figure 1: Quantification of immunoreactive area and object density in 6E10 and** **pGlu-3 Aβ labelled mouse brain.**

Immunoreactive (IR) area in percent and object density in n/mm^2^ in cortex and hippocampus were quantitatively analyzed for 6E10 and pGlu-3 Aβ labelling. 6E10 cortical immunoreactive area (A), 6E10 cortical object density (B), pGlu-3 Aβ cortical immunoreactive area (C), pGlu-3 Aβ cortical object density (D); 6E10 hippocampal immunoreactive area (E), 6E10 hippocampal object density (F), pGlu-3 Aβ hippocampal immunoreactive area (G), pGlu-3 Aβ hippocampal object density (H). Data are mean + SEM, n=3 mice per group. *p*-values are calculated using one-way ANOVA followed by *Dunnett’s* multiple comparison test. ns: not significant; *p<0.05; **p<0.01.


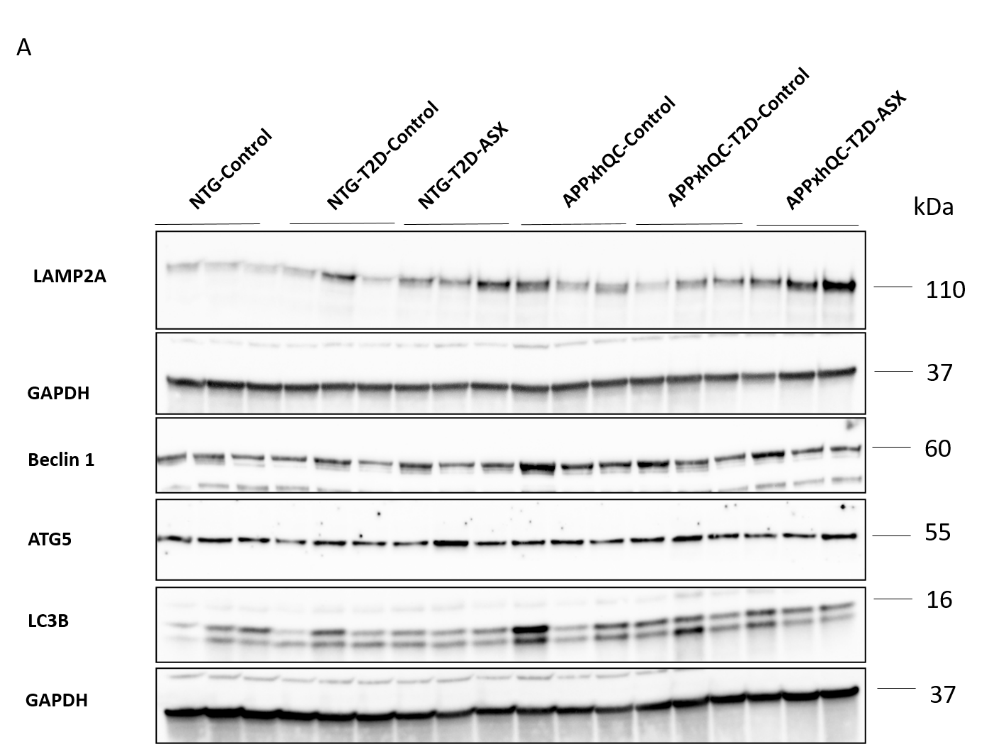


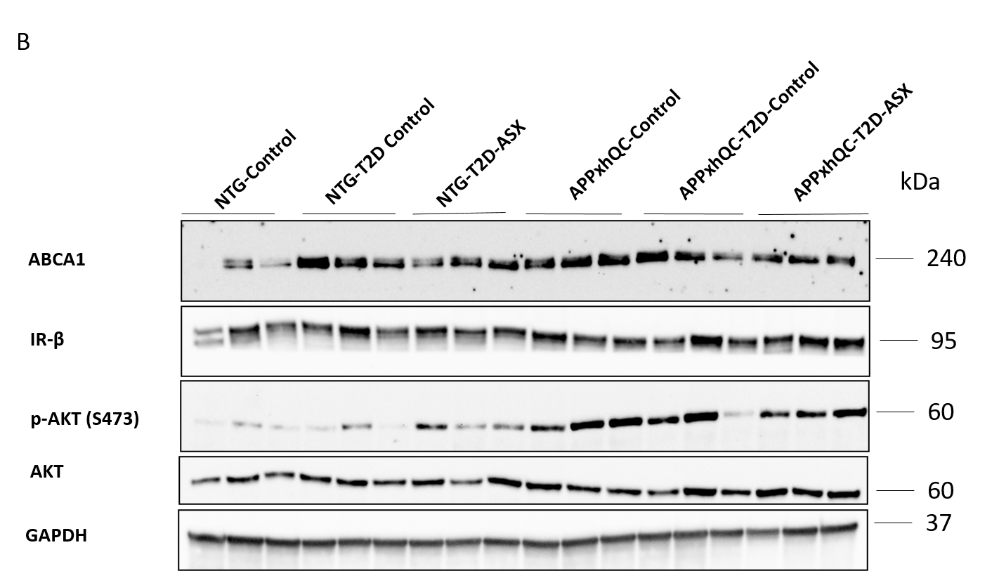


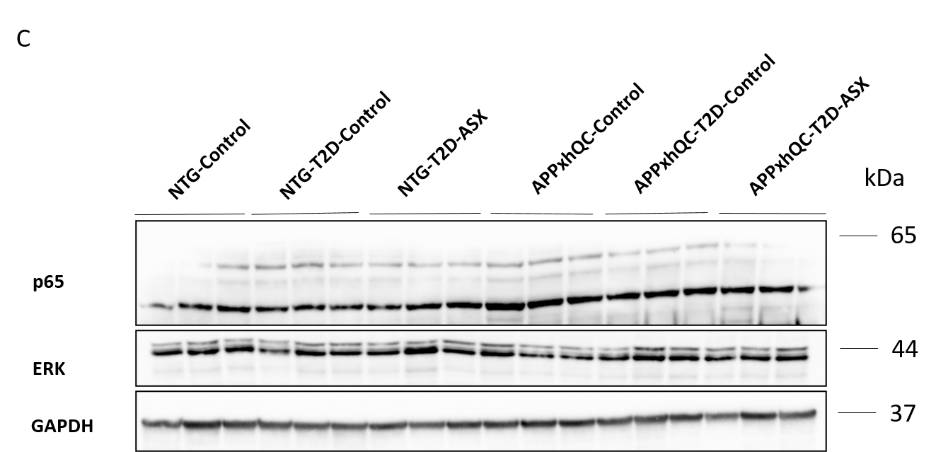


**Supplementary Figure 2: ASX treatment enhances hepatic nutrient sensing signaling in diabetic NTG mice.**

Representative immunoblots for LAMP2A, Beclin1, ATG5, LC3B and GAPDH (A), ABCA1, IR-β, p-AKT, AKT and GAPDH (B) p65 and ERK proteins (C) in the liver of APPxhQC and NTG mice. n=3 mice per group.


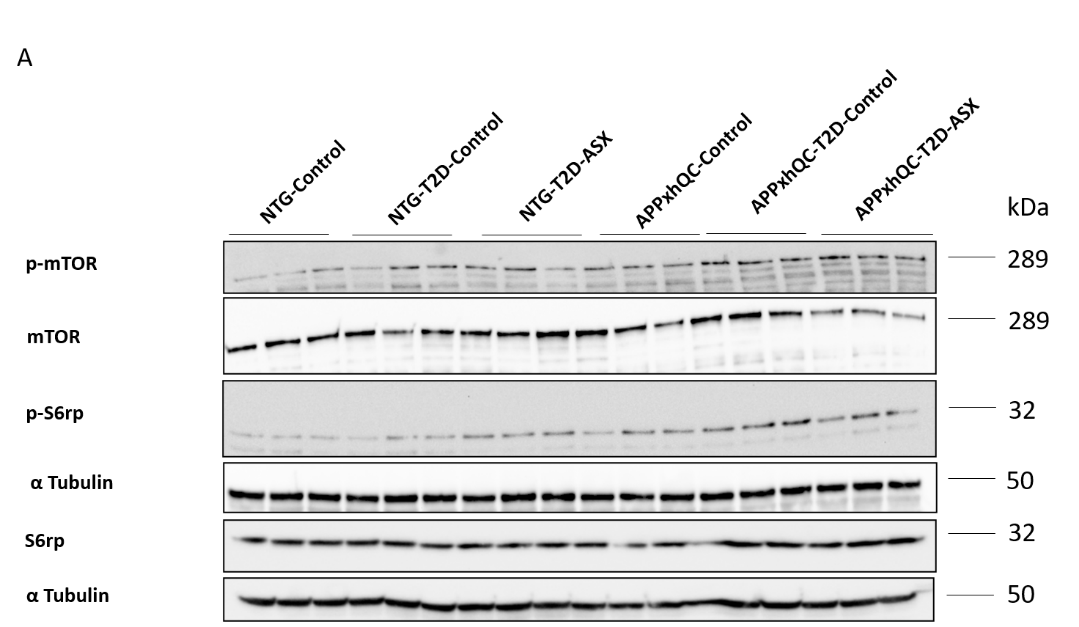


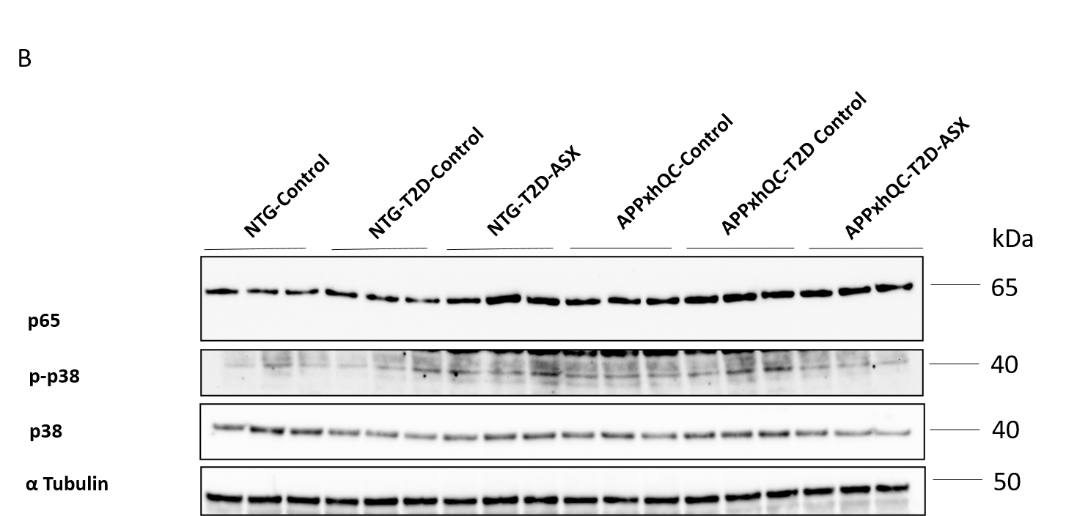


**Supplementary Figure 3: ASX treatment enhances cerebral mTORC1 and MAPKinase signaling in diabetic NTG mice.**

Representative immunoblots for p-mTOR, mTOR, p-S6rp, S6rp and α-tubulin (A) p65, p-p38, p38 and α-tubulin (B) in the brain of APPxhQC and NTG mice. n=3 mice per group.
